# Intestinal T lymphocyte homing is associated with gastric emptying and epithelial barrier function in critically ill: a prospective observational study

**DOI:** 10.1186/s13054-017-1654-9

**Published:** 2017-03-22

**Authors:** Christian Greis, Zohal Rasuly, Rolf A. Janosi, Lambros Kordelas, Dietrich W. Beelen, Tobias Liebregts

**Affiliations:** 1 Department of Bone Marrow Transplantation, University of Duisburg-Essen, University Hospital Essen, West German Cancer Center, Hufelandstr. 55, Essen, 45122 Germany; 2 Department of Cardiology, University Hospital Essen, Essen, Germany

**Keywords:** Gastric emptying, Gut homing, Mucosal barrier, T lymphocytes, Intensive care, Critically ill

## Abstract

**Background:**

Impaired gastric emptying is common in critically ill patients. Intestinal dysmotility, a major cause of feed intolerance, may foster infectious complications due to mucosal barrier disruption. However, little is known about gut-directed immune activation, intestinal barrier function and its association with impaired gastric emptying in critically ill patients at ICU admission.

**Methods:**

We conducted a prospective observational study at two tertiary care medical ICUs. Fifty consecutive patients needing invasive mechanical ventilation were recruited within 24 h of ICU admission, prior to any nutritional support. The acute physiology and chronic health evaluation (APACHE) II score, the sequential organ failure assessment (SOFA) score and the multiple organ dysfunction score (MODS) were used to assess illness severity and multiple organ dysfunction. Gastric emptying was assessed by paracetamol absorption test. Peripheral blood mononuclear cells were freshly isolated and cultured for 24 h, and TNF-α, IL-1β and IL-10 measured in cell culture supernatants and in serum by ELISA. The intestinal epithelial barrier was assessed, quantifying serum concentrations of intestinal fatty acid binding protein (I-FABP), ileal bile-acid binding protein (I-BABP) and zonulin-1 by ELISA. Small bowel homing T lymphocytes (CD4+ α4β7 + CCR9+) were analyzed by flow cytometry. The Mann-Whitney test and Spearman correlation were used in statistical evaluation.

**Results:**

CD4 + α4β7 + CCR9+ T lymphocytes were inversely correlated with gastric emptying. Patients with delayed gastric emptying at ICU admission (n = 35) had significantly higher serum and PBMC-induced TNF-α and IL-1β and increased intestinal barrier disruption reflected by higher I-FABP, I-BABP and zonulin-1. Patients who died in the ICU had significantly impaired gastric empting at admission compared to ICU survivors. No differences were observed in APACHE II, SOFA or MODS in patients with delayed gastric emptying compared to patients with normal gastric emptying.

**Conclusions:**

Exaggerated CD4 + α4β7 + CCR9+ T lymphocyte homing with increased pro-inflammatory cytokine release and intestinal epithelial barrier disruption are associated with delayed gastric emptying. This is not simply due to differences in overall severity of illness at ICU admission and may represent a pathophysiological mechanism of gut-directed immune activation leading to impaired barrier function in the critically ill.

## Background

Enteral nutrition via the gastrointestinal tract is accepted to improve health outcomes in critically ill patients [1]. Gastrointestinal motility disorders, in particular impaired gastric emptying frequently impede enteral nutrient delivery. The advantages of enteral nutrition include a decrease in infectious complications and mucosal barrier protection against bacterial translocation [2, 3]. The highest incidence of delayed gastric emptying in the critically ill has been reported in patients with sepsis [4].

The intestine participates in the inflammatory response locally with cytokine production and leukocyte recruitment. To perform their immunological functions, T lymphocytes must exit the blood and enter into different tissues in the body. Recruitment of leukocytes during an inflammatory response involves the expression and activation of adhesion molecules. CD4+ T lymphocytes responding to the antigen in intestinal lymph nodes start to express high levels of the integrin α4β7 and the chemokine receptor CCR9. The intestinal ligand of CCR9 is CCL25 and it is mainly expressed in the mucosal epithelium of the small intestine. Thus, co-expression of α4β7 and CCR9 delineates a subset of T lymphocytes preferentially migrating to the lamina propria of the small intestine [5].

While we have previously identified association between CD4 + α4β7 + CCR9+ T lymphocytes and gastric emptying [6], we hypothesized that CD4 + α4β7 + CCR9+ T lymphocytes are associated with enhanced pro-inflammatory cytokine secretion, intestinal barrier disruption and delayed gastric emptying in the critically ill. While assessment of gastrointestinal permeability by the frequently applied sugar absorption tests are not reliable in ICU patients [7] we choose to quantify plasma zonulin, ileal bile acid binding protein (ileal bile-acid binding protein) and intestinal fatty acid binding protein (intestinal fatty acid binding protein). Scintigraphy is considered the gold standard for evaluation of gastric emptying. Unfortunately, all currently available tests for the assessment of gastrointestinal motility, including scintigraphy, have limited value in the ICU setting. Major obstacles include lengthy procedures, the need to transfer patients, lack of accuracy in multi-organ dysfunction, costs and availability [8]. In the present prospective observational study, we have chosen the paracetamol absorption test for the assessment of gastric emptying as it is a feasible and reliable technique in the ICU setting [9, 10].

## Methods

### Study population

Fifty consecutive critically ill patients with immediate need of invasive mechanical ventilation were recruited within 24 h of ICU admission. A blood sample was drawn from these patients for cell isolation, serum cytokine ELISA and t0 paracetamol concentration measurement followed by immediate application of paracetamol for assessment of gastric emptying. None of these patients received any enteral or parenteral nutritional support within 24 h of ICU admission. Patients receiving any pro-kinetic drug within 7 days prior to admission were not included.

The leading cause of ICU admission was sepsis, acute respiratory failure or cardiac failure (Table 1). Patients with an underlying malignancy and relapse or disease progression or hematopoietic stem cells recipients with acute graft versus host disease were not included. No surgical or postoperative patients were included. The study was approved by the University Hospital Essen Human Ethics Committee and conducted in accordance with the World Medical Association Declaration of Helsinki. Informed consent was obtained from each patient’s next of kin. In addition eligibility for study participation was confirmed by an independent physician.

**Table 1:** Patient characteristics

	Delayed gastric emptying (n = 35)	Normal gastric emptying (n = 15)
Demographics		
Age (years)	58.4 ± 15.2	62.1 ± 11.4
Gender (male/female), *n*	17/18	8/7
Body mass index (kg/m^2^)	27.6 ± 7.3	27.3 ± 6.2
Admission diagnosis, *n* (%)		
Sepsis	13 (37)	7 (47)
Acute respiratory failure	12 (34)	5 (33)
Cardiac failure	10 (29)	3 (20)
Underlying condition		
Hematologic malignancies, *n* (%)	17 (49)	7 (47)
➢ Acute myeloid leukemia	11 (64)	4 (58)
➢ Lymphoma (B cell)	2 (12)	1 (14)
➢ Myelodysplasia	2 (12)	1 (14)
➢ Myeloproliferative disease	1 (6)	1 (14)
➢ Multiple myeloma	1 (6)	0
Solid tumors	5 (14)	4 (26)
HSCT	4 (11)	1 (7)
Cardiac ischemia	6 (17)	2 (13)
Solid organ transplant	3 (9)	1 (7)
Renal function, *n* (%)		
Failure	16 (46)	6 (40)
Renal replacement therapy	9 (26)	5 (33)
Biochemistry		
Serum bilirubin level (mg/dl)	1.4 ± 1.8	0.9 ± 0.7
White blood cell count (/nl)	8.8 ± 6.4	10.8 ± 7.1
Serum creatinine (mg/dl)	1.2 ± 1.4	0.9 ± 1.1
Glycemic control		
Blood glucose level (mg/dl)	124.0 ± 39.0	143.3 ± 24.1
Insulin (IE/h)	2.6 ± 5.3	1.8 ± 2.2
Medications, *n* (%)		
Sufentanil	35 (100)	15 (100)
Midazolam	26 (74)	11 (73)
Propofol	9 (26)	4 (27)
Vasoactive drugs	23 (66)	12 (80)
Inotropes	10 (29)	5 (33)
Mechanical ventilation		
Pao_2_/Fio_2_ ratio	213.2 ± 76.4	229.1 ± 96.2
Peak inspiratory pressure (mmHg)	22.6 ± 4.9	23.3 ± 3.2
Positive end-expiratory pressure (mmHg)	7.8 ± 2.9	7.7 ± 2.8
ICU length of stay (days)	33.8 ± 30.1	24.7 ± 18.1

Results are presented as mean ± SD or number (percentage). *HSCT* allogeneic hematopoietic stem cell transplantation, Pao_2_/Fio_2_ partial arterial oxygen pressure/fraction of inspired oxygen

### ICU severity scores

Severity of illness was assessed by the acute physiology and chronic health evaluation (APACHE) II score and the severity of multiple organ dysfunction was assessed by sequential organ failure assessment (SOFA) and the multiple organ dysfunction score (MODS).

### Gastric emptying

Gastric emptying was assessed by the paracetamol absorption test [9]. Briefly, 2 g paracetamol was administered with 20 ml water via a nasogastric tube. Blood samples were drawn a time (t) = 0, 15, 30, 60, 90 and 120 minutes to measure paracetamol levels determined by the enzymatic degradation method. The area under the concentration curve from 0 to 60 minutes (AUC60) was used as the measure of gastric emptying. An AUC <600 min*mg/l was considered as delayed gastric emptying [9].

### Cell isolation and culture conditions

For the following cell culture and ELISA experiments investigators were blinded to study subject details, including diagnosis, age and gender. Peripheral blood mononuclear cells (PBMC) were freshly isolated by density gradient centrifugation. Diluted blood (1:2 in RPMI 1640 medium) was layered onto Ficoll-Hypaque (Sigma, Castle Hill, NSW, Australia) and centrifuged at 400 g for 15 minutes. PBMC were washed twice with sterile PBS and viability was assessed by trypan blue exclusion. PBMC were re-suspended to 1 × 10^6^ cells/ml in complete medium (RPMI 1640 medium (Gibco, Karlsruhe, Germany), supplemented with 10% fetal calf serum, 100 U/ml penicillin, 0.1 mg/ml streptomycin and L-Glutamine). PBMC were cultured in 24-well plates for 24 h.

### ELISA

Serum and cell-free culture supernatants were collected, diluted in the supplied dilution buffer or 1:1 in RPMI medium and stored at -80 °C until assayed. TNF-α, IL-1β and IL-10 were quantified using ELISA kits (eBioscience, San Diego, CA, USA) according to the manufacturer’s instructions with minor modifications. Human I-FABP (Hycultbiotech, Beutelsbach, Germany), Zonulin and I-BABP (Cusabio, Wuhan, China) were analyzed utilizing commercially available ELISA kits. Optical density was measured at a wavelength of 450 nm and a reference wavelength of 590 nm. Density values were linearly correlated with the concentrations of test standards.

### Flow cytometry

To identify small bowel homing T lymphocytes freshly isolated PBMC (10^5^) were labeled with previously determined optimal concentrations of monoclonal antibodies directed against human CD3 (APC; eBioscience), CD4 (PerCp; eBioscience), CD49d (α4-Integrin) (FITC; BioLegend), β7-Integrin (PE; BioLegend) and CCR9 (Alexa647; BioLegend). Matching IgG1 and IgG2a conjugated antibodies served as isotype controls. Cells were incubated with the antibodies for 30 minutes on ice and washed three times in FACS buffer between each step. Nonspecific binding of antibodies was minimized by 10-minute incubation of cell suspensions with pooled human serum (10%) in fluorescence-activated cell sorting (FACS) buffer before addition of the first antibody. After washing twice, cells were re-suspended in 100 μl of 1% paraformaldehyde in PBS and analyzed utilizing a Beckman Coulter Navios. Lymphocyte populations were gated based on forward scatter/side scatter properties. A total of 3 × 10^4^ events were routinely collected and analyzed.

### Data analysis

The non-parametric Wilcoxon rank-sum test was used to evaluate differences between groups according to normal and delayed gastric emptying reflected by paracetamol absorption below or above 600 min*mg/l and ICU survival. Data are expressed as median, 95% confidence interval; throughout the manuscript, *n* represents the number of subjects. The relationship between CD4 + α4β7 + CCR9+ T lymphoctyes and paracetamol absorption was assessed by Spearman rank correlation. In all cases *p* values <0.05 were considered significant. SPSS Version 12 (Statistical Package for Social Sciences, Chicago, I, USA) was used for the statistical analysis.

## Results

### Patient characteristics

Patient demographics are displayed in Table 1. Dosage of vasoactive drugs, inotropes and sedative or analgesic agents did not differ significantly between patients with and without delayed gastric emptying. No differences were observed in age, gender, body mass index (BMI), biochemical measurements, glycemic control or mechanical ventilation parameters. All patients were initially ventilated with biphasic positive airway pressure. Patients with and without delayed gastric emptying did not differ in the incidence of acute renal failure or need of continuous renal replacement therapy.

### Gastric emptying

**Fig. 1:**
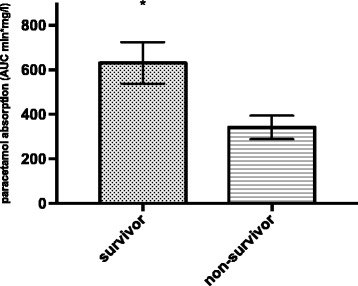
Paracetamol absorption in ICU survivors compared to non-survivors. Patients who died in the ICU had significantly lower paracetamol absorption (*p* = 0.004). *AUC* area under the curve

There were 35 patients (70%) with impaired paracetamol absorption, which was used as a measure of delayed gastric emptying at ICU admission. Gastric emptying was significantly (*p* = 0.004) different (Fig. 1) in patients who died in the ICU (AUC 323.8 ± 48.1) compared to ICU survivors (647.4 ± 93.5). Overall mortality in the ICU was 50%.

### ICU severity scores

**Fig. 2:**
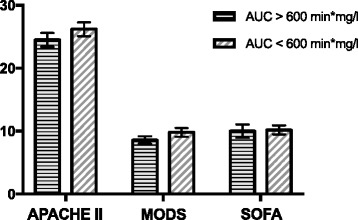
Severity of illness reflected by the acute physiology and chronic health evaluation (*APACHE*) II score, severity of multiple organ dysfunction assessed by the sequential organ failure assessment (*SOFA*) or the multiple organ dysfunction score (*MODS*) in patients with delayed (area under the curve (*AUC*) <600 min*mg/l) and normal gastric emptying (AUC ≥600 min*mg/l)

There were no significant differences in the APACHE II (27.5, 95% CI 18.0–34.0 vs. 24.0, 95% CI 19.0–30.0; *p* = 0.180), SOFA (10.0, 95% CI 5.0–17.0 vs. 10.0, 95% CI 4.0–19.0; *p* = 0.840) or MODS (9.0, 95% CI 5.0–13.0 vs. 9.0, 95% CI 4.0–17.0; *p* = 0.380) in patients with delayed gastric emptying compared to patients with normal gastric emptying (Fig. 2).

### Small bowel homing T lymphocytes

**Fig. 3:**
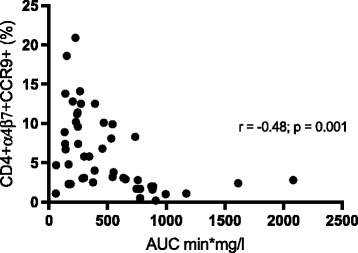
Percentage of CD4 + α4β7 + CCR9+ T lymphocytes was inversely correlated (*r* = -0.5; *p* = 0.001) with gastric emptying as reflected by paracetamol absorption (area under the curve (*AUC*) min*mg/l)

CD4 + α4β7 + CCR9+ T lymphocytes were inversely correlated (*r* = -0.5; *p* = 0.001) with gastric emptying as reflected by paracetamol absorption (Fig. 3).

### Cytokines

**Fig. 4:**
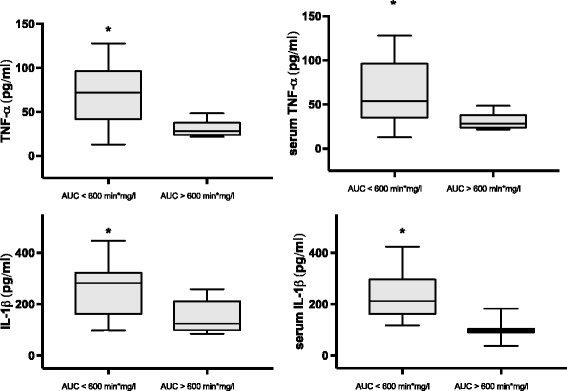
TNF-α and IL-1β (pg/ml) in peripheral blood mononuclear cell culture supernatants and serum of patients with delayed gastric emptying (area under the curve (*AUC*) <600 min*mg/l) and normal gastric emptying (AUC ≥600 min*mg/l). *Error bars* range, *bold line* median, *box* 5th–95th centiles)

Patients with delayed gastric emptying had significantly enhanced serum TNF-α (54.0 pg/ml, 95% CI 13.0–127.9 vs. 28.2 pg/ml, 95% CI 21.8–48.4; *p* = 0.022) and IL-1β (98.5 pg/ml, 95% CI 37.0–182.6 vs. 212.2 pg/ml, 95% CI 91.0–548.6; *p* = 0.005) compared to patients with normal gastric emptying. Release of PBMC-mediated TNF-α (86.5 pg/ml, 95% CI 17.4–342.2 vs. 41.5 pg/ml, 95% CI 31.9–98.6; *p* = 0.020) and IL-1β (280.4 pg/ml, 95% CI 94.0–412.5 vs. 124.6 pg/ml, 95% CI 85.3–247.3; *p* = 0.001) was significantly augmented in patients with delayed gastric emptying. No significant differences in IL-10 were observed (Fig. 4).

### Mucosal integrity

**Fig. 5:**
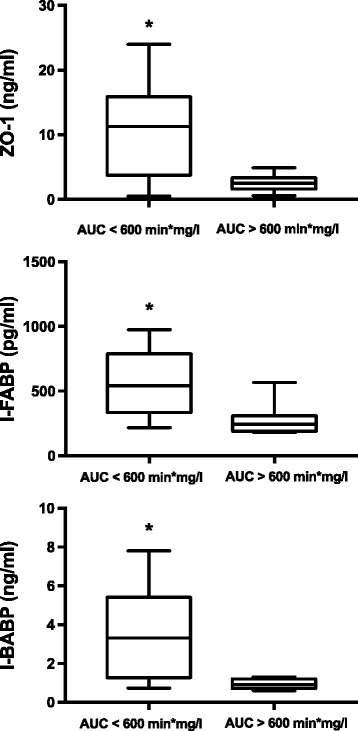
Serum concentration of zonulin-1 (*ZO-1*), intestinal fatty acid binding protein (*I-FABP*) and ileal bile acid binding protein (*I-BABP*) in patients with delayed gastric emptying (area under the curve (*AUC*) <600 min*mg/l) and normal gastric emptying (AUC ≥600 min*mg/l). Error bars, bold line and box respectively represent range, median and 5th-95th centiles *Error bars* range, *bold line* median, *box* 5th–95th centiles)

Delayed gastric emptying was associated with significantly enhanced zonulin (10.4 ng/ml, 95% CI 0.5–59.0 vs. 2.5 ng/ml, 95% 0.6–4.9; *p* = 0.020), I-BABP 3.7 ng/ml, 95% CI 0.7–7.8 vs. 1.0 ng/ml, 95% CI 0.6–1.3; *p* = 0.015) and I-FABP (592.0 pg/ml, 95% CI 218.0–975.0 vs. 244.5 pg/ml, 95% CI 180.0–567.0; *p* = 0.009) (Fig. 5).

## Discussion

The inability of enteral feeding due to delayed gastric emptying frequently necessitates total parenteral nutrition. Total parenteral nutrition itself leads to mucosal inflammatory response resulting in a loss of epithelial barrier function [2], further contributing to intestinal motility disturbances. Increased intestinal permeability predicts the development of multiple organ failure and early enteral nutrition is a key factor for preservation of gut barrier function reducing the risk of organ failure [11]. Furthermore, enterocyte damage has been reported to be associated with ICU mortality [12].

While in most studies feed-intolerant patients or patients receiving parenteral nutrition have been examined, we investigated immune activation with epithelial barrier dysfunction and gastric emptying at admission prior to any enteral or parenteral nutritional support. Zonulin increases intestinal permeability in the epithelia of the small intestine and increased plasma zonulin has been reported in sepsis [13]. I-FABP is particularly expressed in the jejunum and if released into plasma indicates enterocyte destruction [14, 15], while I-BABP is exclusively present in the ileum [16]. We identified increased Zonulin, I-BABP and I-FABP levels in patients with delayed gastric emptying, indicating marked differences in upper gastrointestinal mucosal barrier integrity at the onset of critical illness.

In our study patients with delayed gastric emptying had significantly higher CD4 + α4β7 + CCR9+ T lymphocytes compared to patients with normal gastric emptying at ICU admission. Indeed CCR9 is required for effector CD4+ T lymphocyte entry into small intestinal lamina propria [17]. Under inflammatory conditions intra-epithelial lymphocytes alter barrier function, consequently enhancing intestinal permeability and further enhancing chemokine production [18]. In particular, IFABP and IBAP were inversely correlated with gastric emptying, indicating small bowel immune activation with intestinal barrier alterations associated with gastric emptying. There is further evidence linking intestinal immune activation with CD4 + α4β7 + CCR9+ T lymphocytes and gastrointestinal motility. The detection of CCR9-expressing T lymphocytes in the bloodstream of patients with post-operative ileus has been reported to potentially reflect dysmotility [19].

Apart from increased CD4 + α4β7 + CCR9+ T lymphocytes, we also identified increased serum cytokine levels and pro-inflammatory cytokines produced in vitro by PBMC. Intestinal dysmotility is known to be accompanied by increased expression of pro-inflammatory cytokines such as TNF-α and IL-1β, followed by infiltration of lymphocytes [20]. Based on animal models, TNF-α and IL-1β are known to directly inhibit gastric emptying [21] and decrease small bowel muscle contractility [22]. The increased release of pro-inflammatory cytokines is followed by infiltration of T lymphocytes and macrophages [23]. Macrophages activated by CD4+ T lymphocytes in the intestine initiate the release of nitric oxide, directly inhibiting smooth muscle cell function [24] consequently contributing to intestinal dysmotility. In a previous study, both cytokines were enhanced in patients with idiopathic gastroparesis even in the absence of an infectious stimulus [6]. Thus, our data clearly suggest an association between increased pro-inflammatory cytokines with delayed gastric emptying in the critically ill. Furthermore, TNF-α is a critical factor for epithelial barrier dysfunction [25]. A significant increase in TNF-α in bowel segments is linked to an impaired epithelial barrier [3] within these segments. We therefore believe that our findings of enhanced epithelial barrier function reflected by increased zonulin, I-BABP and I-FABP in subjects with delayed gastric emptying depict a putative causal link between initial immune activation and gastric dysmotility.

Utilizing the paracetamol absorption test, we found 70% of patients, who were admitted to the ICU and needed immediate invasive mechanical ventilation, had impaired gastric emptying. This is in line with published data showing delayed gastric emptying in 42–81% of ICU patients [26, 27]. While the diagnosis on admission exerts a modest impact on gastric emptying in the critically ill, it appears to be most prominent in sepsis. Indeed, the majority of patients included in the present study were admitted to ICU with an infectious complication leading to sepsis or acute respiratory failure. Against the background of published data demonstrating an association between gastric emptying and severity of illness [4] it could be argued that the observed differences in immune activation and epithelial barrier function parameters between patients with normal and delayed gastric emptying are simply a function of baseline severity of illness. However, we did not observe differences in the APACHE II, MODS or SOFA in our study population.

Other potentially confounding factors include sedation and glycemic control. It has been demonstrated that patients receiving morphine and midazolam are more likely to have slow gastric emptying than those receiving propofol [28]. The majority of the patients included in our study received midazolame and sufentanil, without significant differences between the groups. However, an effect of sedation on the overall rate of delayed gastric emptying cannot be excluded. There is abundant literature on glycemic control in the critically ill and blood glucose concentration could be one of a number of factors affecting gastric emptying [29]. Our patients had comparable blood glucose levels and equal insulin doses to achieve glycemic control. Therefore, a profound effect on gastric emptying cannot be assumed.

It also needs to be acknowledged that the majority of patients in our study had an underlying malignancy. There are some data indicating T lymphocyte dysfunction in patients with hematologic malignancies, potentially differing from the general population of critically ill patients. Different degrees of T lymphocyte dysfunctionality, including impaired proliferation, altered cytokine secretion and altered expression of surface molecules, have been described in various hematologic malignancies. In particular T cell lymphoma, chronic myeloid leukemia and chronic lymphoid leukemia are known to be associated with T lymphocyte dysfunctionality. The invasive spreading ability of leukemic cells may depend on the expression of surface molecules including α4β7-Integrin.

There are limited data on neoplastic cells in T cell lymphoma, not achieving remission, expressing α4β7-Integrin when infiltrating bone marrow [30]. Increased expression of α4β7-Integrin is also observed in hematopoietic stem cell recipients with acute gastrointestinal graft versus host disease [31]. Therefore, it needs to be emphasized that we did not include patients with relapse, disease progression or acute graft versus host disease in this study. In addition, the majority of patients with an underlying hematological malignancy had acute myeloid leukemia, which is not associated with a T lymphocyte proliferation defect or impairment of cytokine secretion [32]. Furthermore patients in remission or patients who have undergone intensive chemotherapy may have reduced CD4+ T lymphocytes but without impairment of proliferation [33]. Only when T lymphocyte counts are very low are the remaining T lymphocytes functionally impaired. Thus, it is important to emphasize that patients in our study had normal white blood cell counts and T lymphocyte populations and therefore do not represent severely neutropenic (<1.500 neutrophils/μl) patients with cancer, whose inclusion could potentially confound the immune activation results. However, the observed ICU mortality of 50% reflects current mortality rates of critically ill patients with cancer [34] and is therefore certainly due to the high percentage of patients in our study who had malignancies.

## Conclusions

To our knowledge this study is the first to identify a link between gastric dysmotility and increased small bowel-directed immune activation and mucosal barrier impairment in ICU patients at the onset of critical illness, in the absence of any nutritional support. Our result suggests that initial immune activation leads to increased release of pro-inflammatory cytokines followed by infiltration of lymphocytes. While cytokines are able to directly impair gastrointestinal motility, lymphocytes may contribute to delayed gastric emptying by activating macrophages releasing nitric oxide as a motility inhibitor. Cytokines also impair intestinal permeability, leading to amplification of the inflammatory response. Thus, this cascading inflammation may drive organ failure including impaired intestinal barrier and motility as an underlying cause of further intestinal dysfunction, regardless of the overall severity of the initial illness.

## Abbreviations

APACHE II: Acute physiology and chronic health evaluation score
AUC60: Area under the concentration curve from 0 to 60 minutes
ELISA: Enzyme-linked immunosorbent assay
FACS: fluorescence-activated cell sorting
HSCT: Allogeneic hematopoietic stem cell transplantation
I-BABP: Ileal bile-acid binding protein
I-FABP: Intestinal fatty acid binding protein
IL: Interleukin
MODS: Multiple organ dysfunction score
PBMC: Peripheral blood mononuclear cells
PBS: phosphate-buffered saline
RPMI: Roswell Park Memorial Institute medium
SOFA: Sequential organ failure assessment
TNF: Tumor necrosis factor
